# Commentary: Association between serum estradiol levels and cognitive function in older women: a cross-sectional analysis

**DOI:** 10.3389/fnagi.2025.1559647

**Published:** 2025-05-20

**Authors:** Jiayi Chen

**Affiliations:** Department of Stomatology, Suzhou Wujiang District Hospital of Traditional Chinese Medicine, Suzhou, Jiangsu, China

**Keywords:** NHANES, neurology, logistic (logit) regression, cross-sectional study, cognitive function

It is a privilege to have had the opportunity to read the article entitled “*Association between serum estradiol levels and cognitive function in older women: a cross-sectional analysis”* (Xu et al., 2024). I commend the authors, Xu et al., for their important contribution to the field of neurology. Their cross-sectional study examines the relationship between serum estradiol levels and cognitive function in older women, an area of research that holds significant relevance for public health. However, after reviewing the article, I have some questions and concerns about the methodology, which I would like to bring to the authors' attention.

First, although the authors provide a detailed flowchart of the data collection process from the NHANES database, there is no mention of how the sample size for this cross-sectional study was calculated. This is an important aspect of research design and would help clarify the robustness of the study's findings (Ritz et al., 2022).

In addition, the NHANES database utilizes an oversampling method to address racial and ethnic imbalances in the U.S. population. However, in this study, I noticed that non-Hispanic White participants represented 78.95% of the sample, while non-Hispanic Black participants accounted for only 8.83%. This imbalance may affect the generalizability of the study's findings, and further clarification on how this was accounted for in the analysis would be appreciated.

I also believe that the Methods and Materials section would benefit from further elaboration on the definitions of covariates such as hypertension and diabetes mellitus (DM). For example, it would be helpful to know how borderline DM was classified in the study and how this classification might have influenced the results.

Another area of concern is the potential collinearity between variables in the logistic regression (LR) model. For instance, the relationship between DM and body mass index (BMI) has been well-established in prior research (Wang et al., 2023), and I am curious about how the authors addressed potential collinearity issues between these and other variables in the LR model. Furthermore, I wonder whether the inclusion of too many variables in the model might lead to overfitting or if too few variables could risk underfitting.

In summary, this study provides valuable insights into the potential association between serum estradiol levels and cognitive function in older women, and it raises important questions for future research. I look forward to the authors' responses to these inquiries.
