# Correction: Commentary: Association between serum estradiol levels and cognitive function in older women: a cross-sectional analysis

**DOI:** 10.3389/fnagi.2026.1806072

**Published:** 2026-03-03

**Authors:** Jiayi Chen

**Affiliations:** Department of Stomatology, Suzhou Wujiang District Hospital of Traditional Chinese Medicine, Suzhou, Jiangsu, China

**Keywords:** NHANES, neurology, logistic (logit) regression, cross-sectional study, cognitive function

In the published article, the title was erroneously given as “Commentary: The letter to editor regarding “Association between serum estradiol levels and cognitive function in older women: a cross-sectional analysis”. The correct title of the article is “Commentary: Association between serum estradiol levels and cognitive function in older women: a cross-sectional analysis”.

The original version of this article has been updated.

